# Medicinal Plants for the Treatment of Gastrointestinal Cancers From the Metabolomics Perspective

**DOI:** 10.3389/fphar.2022.909755

**Published:** 2022-06-27

**Authors:** Wei Guo, Peng Cao, Xuanbin Wang, Min Hu, Yibin Feng

**Affiliations:** ^1^ Department of Pharmacy, Union Hospital, Tongji Medical College, Huazhong University of Science and Technology, Wuhan, China; ^2^ Hubei Province Clinical Research Center for Precision Medicine for Critical Illness, Wuhan, China; ^3^ Laboratory of Chinese Herbal Pharmacology, Department of Pharmacy, Renmin Hospital, Hubei University of Medicine, Shiyan, China; ^4^ School of Chinese Medicine, Li Ka Shing Faculty of Medicine, The University of Hong Kong, Hong Kong, China

**Keywords:** gastrointestinal cancers, medicinal plants (herbal drugs), adjuvant therapeutics, metabolomics, cancer metabolism

## Abstract

Gastrointestinal cancer (GIC), primarily including colorectal cancer, gastric cancer, liver cancer, pancreatic cancer, and esophageal cancer, is one of the most common causes of cancer-related deaths with increasing prevalence and poor prognosis. Medicinal plants have been shown to be a great resource for the treatment of GIC. Due to their complex manifestations of multi-component and multi-target, the underlying mechanisms how they function against GIC remain to be completely deciphered. Cell metabolism is of primary importance in the initialization and development of GIC, which is reported to be a potential target. As an essential supplement to the newest “omics” sciences, metabolomics focuses on the systematic study of the small exogenous and endogenous metabolites involved in extensive biochemical metabolic pathways of living system. In good agreement with the systemic perspective of medicinal plants, metabolomics offers a new insight into the efficacy assessment and action mechanism investigation of medicinal plants as adjuvant therapeutics for GIC therapy. In this review, the metabolomics investigations on metabolism-targeting therapies for GIC in the recent 10 years were systematically reviewed from five aspects of carbohydrate, lipid, amino acid, and nucleotide metabolisms, as well as other altered metabolisms (microbial metabolism, inflammation, and oxidation), with particular attention to the potential of active compounds, extracts, and formulae from medicinal plants. Meanwhile, the current perspectives and future challenges of metabolism-targeting therapies of medicinal plants for GIC were also discussed. In conclusion, the understanding of the action mechanisms of medicinal plants in GIC from the metabolomics perspective will contribute to the clinical application of potential candidates from the resourceful medicinal plants as novel and efficient adjuvant therapeutics for GIC therapy.

## Introduction

### Gastrointestinal Cancer

Gastrointestinal cancer (GIC) defines a series of deadly malignancies in the digestive system of gastrointestinal tract and organs, primarily including colorectal cancer (CRC), gastric cancer (GAC), liver cancer, pancreatic cancer (PCC), and esophageal cancer (EC) (Kuntz et al., 2021). Among all cancer sites, GIC ranks the first in all cancer-related deaths (approximately 28%) and the second in all new cancer cases (approximately 18%) based on the Cancer Statistics 2021, United States (Siegel et al., 2021). In detail, as one of the most commonly diagnosed GIC, CRC occurs in the colon or rectum site of large intestine with the third highest incidence and the leading cause of cancer-related deaths globally (Bray et al., 2018). GAC starts with chronic gastritis and then progresses to gastric atrophy, intestinal metaplasia, dysplasia, and finally develops to adenocarcinoma (Tan and Fielding, 2006). GAC ranks as the fourth most common cancer with the second leading cause of cancer-related deaths all over the world (Van Cutsem et al., 2016). As the commonest primary hepatic cancer, hepatocellular carcinoma (HCC) accounts for approximately 90% of all cases of liver cancer. HCC is the fifth most prevalent malignancy and the third leading cause of cancer-related deaths globally (Siegel et al., 2018). PCC ranks as the seventh deadliest cancer worldwide with a five-year survival rate lower than 5% (McGuigan et al., 2018). It is predicted that PCC would become the second leading cause of cancer-related deaths globally in the next 10 years (Neoptolemos et al., 2018). EC is one of the most aggressive GIC with the sixth leading cause of cancer-related deaths worldwide (Watanabe et al., 2020). Among these five major types of GIC, liver, gastric, and esophageal cancers are common in Asia while pancreatic and colorectal cancers are common in North America and Europe (Arnold et al., 2020). As a greatly costly disease, the overall annual cost for GIC is more than 20 billion dollars all over the world, which causes heavy burden to public health (Aftabi et al., 2021).

As a multifactorial process, the oncogenesis of GIC is induced by both the individual’s gene and associated environment factors (Meng et al., 2018). For example, the incidence of CRC is increased by sedentary lifestyles, smoking, alcohol consumption, and environment (Ji et al., 2018). HCC is predominantly triggered by liver cirrhosis due to exposure to aflatoxin, excessive alcohol consumption, or viral infection (Van Den Bosch and Defreyne, 2012). The mortality of GIC is almost equal to its morbidity, indicating the poor prognosis of GIC. Three conventional therapies, including surgery, radiotherapy, and chemotherapy, remain to be the main current therapeutics for GIC. Early stage of GIC is usually insidious with no obvious symptoms and thus most GIC patients are diagnosed in the middle or late stages, which are not suitable for surgery (Yu et al., 2020). As the main standard therapies for GIC patients who are in middle or late stages, radiotherapy and chemotherapy are still costly and forceless (Dai et al., 2022). In particular, increasing drug resistance and various adverse events occur during radiotherapy and chemotherapy. For example, serious digestive tract reaction, neurotoxicity, and blood toxicity are often observed in GIC patients treated with chemotherapeutic drugs. Therefore, it is extremely urgent to develop new anticancer drugs with high efficacy and few side effects for GIC therapy.

### Metabolomics for Cancer Metabolism

One of the essential hallmarks of cancer cells is the metabolic reprogramming to meet the bioenergetics and biosynthetic requirements for their rapid growth (Pavlova and Thompson, 2016; Martinez-Outschoorn et al., 2017). Compared with normal cells, cancer cells have been indicated to exhibit a remarkably distinct metabolic phenotype, which is characterized by increased energy production, high antioxidant regeneration, enriched macromolecule synthesis, and diminished bioenergetic expenditure (Sharma et al., 2019). In general, cancer metabolism is of primary importance in the tumorigenesis, tumour progression, and metastasis, as well as drug resistance of cancer (Vernieri et al., 2016), and altered metabolites in metabolism pathways including carbohydrate, lipid, amino acid, and nucleotide metabolisms, as well as other altered metabolisms could provide valuable information in developing novel biomarkers for GIC diagnosis and effective targets for GIC treatment. For example, acetate, glutamine, and glycocholate were identified as potential biomarkers with high sensitivity and specificity for the diagnosis of CRC, PCC, and HCC, respectively (Nannini et al., 2020; Gao et al., 2021).

As an essential complement to the newest “omics” sciences, metabolomics focuses on the systematic study of the small exogenous and endogenous molecule metabolites involved in extensive biochemical metabolism pathways of living system with intrinsic or extrinsic factors, which is defined as “metabolome” -the metabolites with an atomic mass less than 1.5 kDa (Johnson et al., 2016; Zampieri et al., 2017). In comparison to other “omics” sciences, including genomics, transcriptomics, and proteomics, in which the modifications of substrates exist in common, metabolomics could offer us a direct and systemic readout on metabolites and tell us accurately what occurred in response to both genetic modifications and pathophysiological stimuli (Aftabi et al., 2021). Besides, the fluctuations at the genome or proteome levels can be amplified at the metabolome level while the members of metabolome are much smaller than those of genome or proteome. Finally, it is cheaper to perform a metabolomic analysis than a genome, a transcriptome or a proteome analysis (Gao et al., 2021).

The advanced analytical chemistry tools and computational means are utilized in metabolomics for high-throughput analysis of metabolome. Mass spectrometry (MS) coupled with liquid chromatography (LC) or gas chromatography (GC), and nuclear magnetic resonance (NMR) spectroscopy consist of the three main analytical chemistry platforms (Hamerly et al., 2015). Each platform has its own pros and cons. There are three main types of methodological means to analyze the small metabolites in metabolomics, including targeted, untargeted, and stable isotope-resolved metabolomics. Metabolomics offers us the valuable information on the potential biomarkers for diagnosing and monitoring the complex diseases and the effective targets as well as the detailed mechanisms for therapeutic intervention (Guo et al., 2016; Wishart, 2016). Considering the importance of metabolic reprogramming in the initialization and development of cancer, metabolomics would contribute to cancer research for the diagnosis and monitoring of cancer, drug discovery, as well as pharmacodynamic and therapeutic evaluation for cancer treatment (Guo et al., 2018; Kaushik and DeBerardinis, 2018; Hoang et al., 2019). To date, metabolomics has also been extensively adopted in the domains of GIC, especially for the biomarker discovery of GIC (Nannini et al., 2020; Yu et al., 2020; Aftabi et al., 2021; Gao et al., 2021).

### Medicinal Plants for Gastrointestinal Cancer Therapy

As a complementary and alternative approach to conventional radiotherapy and chemotherapy gradually accepted in western countries, medicinal plants have been shown to be a huge reservoir for anti-cancer agents due to their low toxicity and multiple biological activities (Wang et al., 2015; Hu et al., 2016). For example, numerous active ingredients from medicinal plants have been identified with antineoplastic activities, such as flavonoids, triterpenes, polysaccharides, and so on (Dai et al., 2022). Adjuvant precursors from the plant origin could enhance the antineoplastic effects and decrease drug resistance of chemotherapy, reduce adverse reactions, as well as improve the immune function in cancer patients (Guo et al., 2020a). Of note, multiple lines of evidences have revealed that considerable amounts of active compounds, extracts, and formulae from medicinal plants exhibit potential antineoplastic effects against different types of GIC (Ying et al., 2018; Aiello et al., 2019; Guo et al., 2019; Huang et al., 2020).

Although medicinal plants have been widely utilized as adjuvant therapeutics for GIC therapy, the underlying anti-GIC mechanisms how they function remain to be completely deciphered due to their own distinct theoretical system with multi-component and multi-target manifestation. In good agreement with the holistic perspective and overall concept of medicinal plants, metabolomics focuses on fluctuations in the overall terminal metabolites and offers a new insight into the efficacy assessment and molecular mechanism investigation of medicinal plants as adjuvant therapeutics for GIC therapy (Nie et al., 2019). The application of metabolomics in biomarker discovery for GIC diagnosis has been reported in various excellent reviews (Nannini et al., 2020; Yu et al., 2020; Aftabi et al., 2021; Gao et al., 2021). However, little reviews have been focused on the efficacy evaluation and molecular mechanism exploration of drugs, especially medicinal plants for GIC therapy based on metabolomics.

**FIGURE 1:**
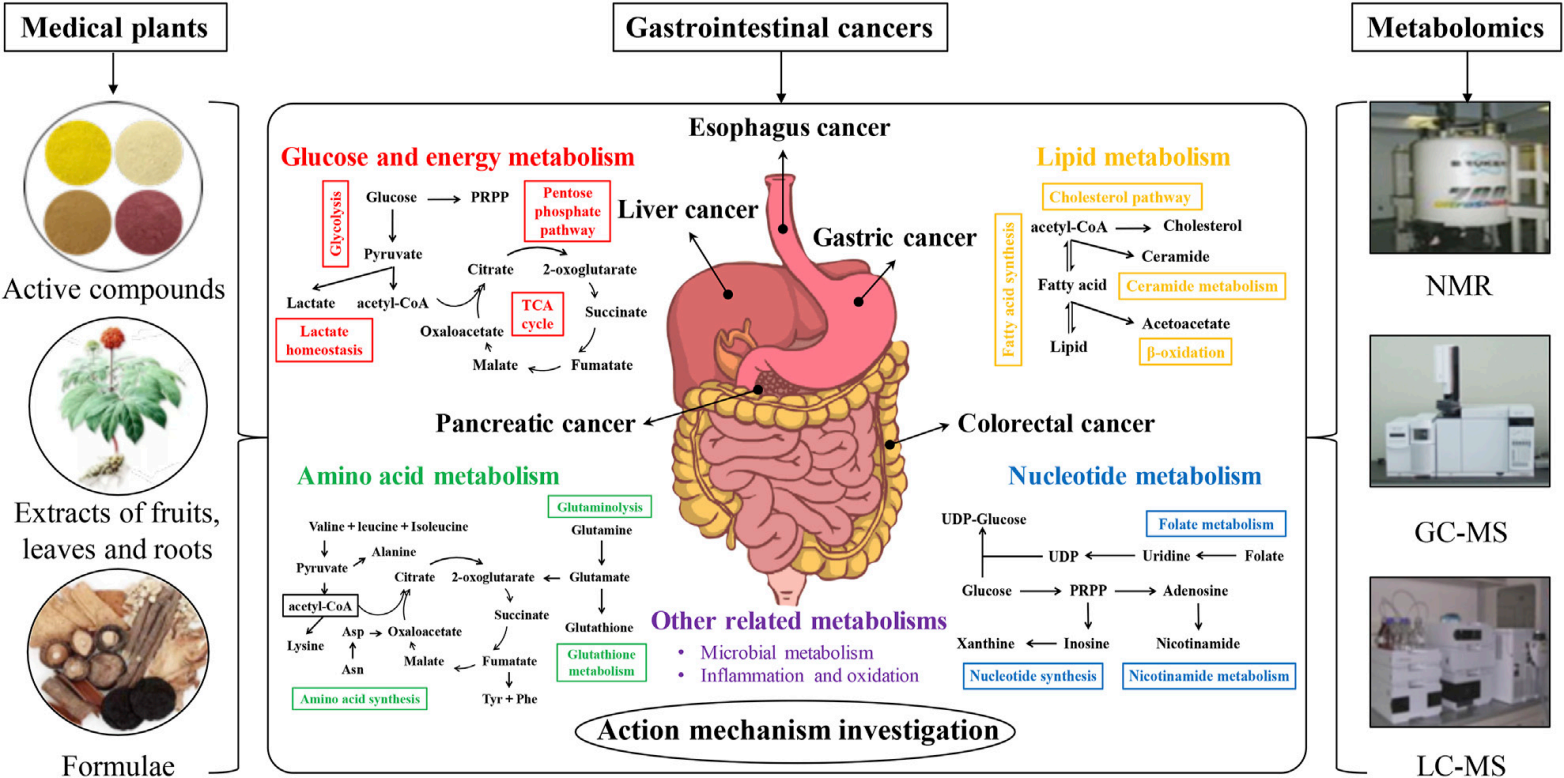
The schematic illustration of action mechanism investigation of medicinal plants in gastrointestinal cancer therapy from the metabolomics perspective.

In this review, the metabolomics investigations on metabolism-targeting therapies for GIC in the recent 10 years were systematically reviewed at five aspects of carbohydrate, lipid, amino acid, and nucleotide metabolisms, as well as other altered metabolisms (microbial metabolism, inflammation, and oxidation), with particular attention to the potential of active compounds, extracts, and formulae from medicinal plants. Figure 1 showed the schematic illustration of action mechanism investigation of medicinal plants in GIC therapy from the metabolomics perspective. The recent metabolomics investigations on anti-GIC therapies of active compounds, extracts, and formulae from medicinal plants were respectively summarized in Tables 1–3. Meanwhile, the current perspectives and future challenges of metabolism-targeting therapies of medicinal plants for GIC were also discussed.

**TABLE 1:** The summary of recent metabolomics investigations on anti-GIC therapies of active compounds from medicinal plants.

References	Active compounds	Medicinal plants	Cancer types	Samples	Methods	Significantly changed metabolites or pathways	Main findings
29448205	6,7-dimethoxy-1,2,3,4-tetrahydro-isoquinoline-3-carboxylic acid (M1)	*M. pruriens* (L.) DC.	Colorectal cancer (CRC)	Serum from DMH-induced CRC rat model *in vivo*	^1^H NMR	The disordered metabolic fluxes in CRC condition, including glycolysis, TCA cycle, gluconeogenesis and phosphatidylinositol metabolisms, were reversed after M1 treatment	M1 regulated the metabolic fluxes to exhibit the anti-CRC potential through the oncogenic signalling inhibition of IL-6/JAK2/STAT3
26160839	Halofuginone (HF)	*D. febrifuga* Lour.	Colorectal cancer (CRC)	HCT116 cells *in vitro*	GC-MS and UPLC-MS	HF treatment exhibited the slower rates in both glycolytic and glucose-derived TCA cycle fluxes	HF modulated Akt/mTORC1 signaling pathways to inhibit glycolysis and TCA cycle in CRC cells
28918937	Koningic acid (KA)	*Trichoderma* fungus	Colorectal cancer (CRC)	HCT116 cells *in vitro*	Integrated LC-MS metabolomics and pharmacogenomics	KA treatment substantially affected glycolytic metabolites with an accumulation of glycolytic intermediates upstream of GAPDH	The basis of targeting the warburg effect could be encoded by molecular principles that extend beyond the status of individual genes
26700591	Geranylgeranoic acid (GGA)	*C*. *longa* L.	Hepatoma	HuH-7 cells *in vitro*	UPLC-TOF-MS	GGA induced a time-dependent decrease of fructose 1,6-diphosphate and increase of fructose 6-phosphate	GGA may shift HuH-7 cells from aerobic glycolysis to mitochondrial OXPHOS through the immediate upregulation of TIGAR and SCO2 protein levels
29187559	Zerumbone	*Z*. *zerumbet* (L.) Roscoe ex Sm.	Hepatocellular carcinoma (HCC)	Huh7, HepG2 and MHCC-LM3 cells *in vitro*	Microarray and 2D NMR	Zerumbone inhibited glycolysis by reducing glucose consumption and lactate production	Zerumbone suppressed HCC by mediating energy metabolism thereby forcing cells to undergo apoptosis and cell cycle arrest
28198625	Curcumin	*C. longa* L.	Hepatocellular carcinoma (HCC)	Serum from DEN-induced HCC mice *in vivo*	GC-MS	Curcumin increased the levels of glucose and fructose as well as decreased the levels of proline and glycine	Curcumin protected the mice from the chemical induced liver injury through suppressing liver cellular metabolism
27077962	Hispidulin	*A. vulgaris* L., *A. montana* L., *and E. littorale* Cabrera	Hepatocellular carcinoma (HCC)	Urine from mice bearing H22 cells *in vivo*	UHPLC-QTOF-MS	Most of the potential biomarkers mediated by hispidulin were associated with TCA cycle	These potential biomarkers underpinned the metabolic pathways, which were perturbed in HCC mice model
32660149	Berberine	*C. chinensis* Franch.	Hepatocellular carcinoma (HCC)	MHCC97L cells *in vitro* and tumor from orthotopic HCC implantation murine model *in vivo*	GC-MS	The metabolic fluctuation induced by berberine was related to glucose-alanine cycle	Berberine suppressed the alanine-glucose conversion *via* GPT1 and blocked ATP production and thus inhibited HCC growth
27416811	Physapubenolide (PB)	*P. pubescens* L.	Hepatocellular carcinoma (HCC)	HepG2 cells *in vitro* and tumor and plasma from a mouse-xenograft model bearing H22 cells *in vivo*	GC-MS	PB mediated the metabolic pattern *via* decreasing lactate production	PB showed anti-HCC activity through suppression of glycolysis *via* the Akt-p53 pathway
28916726	β-Lapachone	*H. impetiginosus* (Mart. ex DC.) Mattos	Pancreatic ductal adenocarcinoma (PDAC)	MiaPaCa2 cells *in vitro*	^1^H NMR and GC-MS	A decrease of nicotinamide adenine dinucleotide-sensitive pathways, such as TCA cycle and glycolysis, was found after β-lapachone treatment	Targeting NQO1 may sensitize the treatment of β-lapachone
32692565	Berberine	*C. chinensis* Franch.	Pancreatic cancer (PCC)	Panc-1 cells and hTERT-HPNE cells *in vitro*	RNA-sequencing and LC-TQ-MS	Berberine strongly dysregulated the energy metabolism of PCC cells *via* targeting citrate metabolism	The cell metabolomics methodology contributed to rapidly investigate biochemical functions of natural products
26859520	Flexibilide	*S. flexibilis*	Colon cancer (CC)	HCT-116 cells *in vitro*	UPLC-QTOF-MS	The significant decrease of phosphocholine and PC and increase of LysoPC were observed after flexibilide treatment	The downregulation of PC biosynthesis pathway contributed to the therapeutic effects of flexibilide against CC
28496003	Peiminine	*F. thunbergii* Miq.	Colorectal cancer (CRC)	HCT-116 cells *in vitro*	UPLC-MS and GC-MS	Several metabolites, including oleate (18:1n9), lignocerate (24:0), glucose, and glutamine were observed to alter after peiminine treatment	The regulation of lipids, carbohydrates, and amino acids contributed to the therapeutic effects of peiminine on CRC
31301538	Ilexgenin A (IA)	*I. hainanensis* Merr.	Colorectal cancer (CRC)	Plasma from AOM/DSS-induced CRC mice model *in vivo*	UHPLC-Q-TOF	IA reversed phospholipid metabolism, glycerophospholipid catabolism, and arachidonic acid metabolism	IA reprogramed lipid metabolism *via* HIF-1α/SREBP-1 to prevent early colonic carcinogenesis
34062256	Psoralen packed in polymer lipid nanoparticles (PSO-PLNs)	*P. corylifolia* L.	Hepatocellular carcinoma (HCC)	Serum and urine from mice engrafted with HepG2 cells resistant against doxorubicin *in vivo*	LC-MS	PSO-PLNs had stronger degree of regression of metabolites including α-linolenic acid, retinoic acid, pantothenic acid, hyaluronic acid, and hippuric acid than psoralen	Psoralen reversed drug resistance of HCC cells, which could be enhanced by encapsulation in polymer lipid nanoparticles
30068874	Celastrol	*T. wilfordii* Hook.f.	Colon cancer (CC)	HCT116 cells *in vitro*	UPLC-MS	The levels of lipid markers, amino acids, and carnitine were significantly altered after celastrol treatment	Further targeted metabolite analysis characterized tryptophan as the key biomarker
32869425	Oridonin	*I. rubescens* (Hemsl.) H.Hara	Esophageal cancer (EC)	TE1 cells *in vitro*	UPLC-MS/MS	Oridonin administration altered the levels of glutamate, 5-oxoproline, and gamma-glutamyl amino acids (gamma-glutamyl valine and gamma-glutamyl leucine)	Oridonin could suppress the gamma-glutamyl cycle to induce ferroptosis to exert its anti-EC activity
33476976	Shikonin	*L. erythrorhizon* Siebold & Zucc.	Colon cancer (CC)	SW480 cells *in vitro*	Transcriptomics and UHPLC-MS	The regulation of purine and pyrimidine metabolisms, urea cycle, and arginine biosynthesis contributed to the anti-CC activity of shikonin	A systematic metabolic perspective in the beneficial effects of shikonin might lay a foundation for further research on shikonin in CC
29651531	Glaucocalyxin A (GLA)	*R. japonica* (Burm.f.) H.Hara	Hepatocellular carcinoma (HCC)	SMMC7721 cells *in vitro*	LC-MS and GC-MS	GLA treatment suppressed amino acid metabolism and increased the metabolisms of sphingolipid, pyrimidine, and purine	The systemic metabolic alterations triggered by GLA treatment inhibited HCC
33072135	Berberine	*C. chinensis* Franch.	Colorectal cancer (CRC)	Stool from AOM/DSS-induced CRC mice model *in vivo*	16S rRNA sequencing and ^1^H NMR	Berberine mediated metabolic fluctuations in feces *via* regulating the metabolites of amino acid metabolism, SCFA metabolism, and glycometabolism, which were the products of interactions between the host and gut microbiota	Berberine induced changes in metabolome and microbiota in CRC, which could provide a novel insight into the anti-CRC effects of berberine
28674386	Caffeic acid (CAA) and chlorogenic acid (CHA)	*L. japonica* Thunb.	Hepatocellular carcinoma (HCC)	Serum from DEN-induced HCC rat model *in vivo*	16S rRNA sequencing, LC-MS and GC-MS	Both CAA and CHA treatments reversed 28 metabolites	The levels of bilirubin, ethanolamine, L-tyrosine, and L-methionine were linked to the increased level of *Rumincoccaceae UCG-004* and decreased levels of *Lachnospiraceae incertae sedis* and *Prevotella 9*
31814271	Nanoparticle with ginsenoside Rg3 (NpRg3)	*P. ginseng* C.A.Mey	Hepatocellular carcinoma (HCC)	Serum and liver tissue samples from DEN-induced spontaneous HCC mice model *in vivo*	16S rRNA sequencing and HPLC-MS	NpRg3 treatment increased free fatty acids, but diminished urea and 3-indolepropionic acid	NpRg3 remodeled the network imbalance between metabolism and gut microbiota, which contributed to inhibit HCC development and metastasis
32531676	Solasonine	*S. melongena* L.	Hepatocellular carcinoma (HCC)	HepG2 cells *in vitro*	LC-MS	Solasonine regulated glutathione metabolism *via* glutathione peroxidase 4 and glutathione synthetase.	Solasonine induced HCC cell ferroptosis by the destruction of the glutathione redox system

**TABLE 2:** The summary of recent metabolomics investigations on anti-GIC therapies of extracts from medicinal plants.

References	Medicinal plant extracts	Cancer types	Samples	Methods	Significantly changed metabolites or pathways	Main findings
28744216	*C. nitidissima* C.W.Chi (CNC)	Colorectal cancer (CRC)	Intestine, kidney and spleen from AOM/DSS-induced CRC mice model *in vivo*	^1^H NMR	CNC extracts could strongly recover the disordered metabolic profiling to the normal status, such as glycolysis, glutamate, and glutamine metabolisms	The butanol fraction exhibited a better efficacy against CRC than the water-soluble fraction of CNC
26186142	Silymarin	Hepatoma	Huh7TLR3 cells *in vitro*	Transcriptional profiling and GC-MS	Silymarin suppressed the glycolysis, TCA cycle, and amino acid metabolism	The attenuated metabolic disorders by silymarin contributed to its antineoplastic activity against hepatoma
27369806	*R. Paridis* Saponins (RPSs)	Hepatocellular carcinoma (HCC)	Serum from HCC mice model bearing H22 cells *in vivo*	^1^H NMR	RPSs diminished the serum levels of glutamine, N-acetyl amino acid, acetate, and lactate	RPSs were potential anti-HCC herb extracts *via* suppressing the glutamine metabolism, lipogenesis, and aerobic glycolysis
25712450	Nutmeg	Colon cancer (CC)	Serum from mice harboring adenomatous polyposis coli gene mutation-induced colon cancer model *in vivo*	UPLC-ESI-QTOF-MS	The lipid metabolism was regulated by the treatment of nutmeg extract *via* diminishing four uremic toxins formed from the gut microbiota	The regulation of lipid metabolism and gut microbiota may be an effective therapy for CC
32657580	Peroxidase from foxtail millet bran (FMBP)	Colorectal cancer (CRC)	Serum from AOM/DSS-induced colitis-associated carcinogenesis mice model *in vivo*	UPLC-Triple/TOF-MS	FMBP predominantly decreased the levels of PC and PE related to GPL metabolism	FMBP was a potential preventive and therapeutic extract to blockade the GPL metabolism for CRC
30587039	*B. Sinularia* sp. *(BS)*	Hepatocellular carcinoma (HCC)	Hep 3B cells *in vitro*	MS	The levels of ceramide and sphingolipids were regulated by BS extract	BS extract showed its anti-HCC property *via* the regulation of disordered lipids
27775667	Polyphenols extracted from chestnut shell (PECS)	Hepatocellular carcinoma (HCC)	HepG2 cells *in vitro*	^1^H NMR	PECS were shown to modulate the levels of some amino acids	The regulation of amino acids may account for the anti-HCC property of PECS
30448539	Annonaceous acetogenins (ACGs)	Hepatocellular carcinoma (HCC)	SMMC 7721 cells *in vitro*	UFLC-ESI-Q-TOF-MS	ACGs treatment could regulate the metabolisms of proline, arginine, glutathione, and sphingolipid, which further reversed the resistance of SMMC 7721 cells to adriamycin	Metabolic pathway analysis combined with stoichiometry analysis could be a potential tool to understand MDR mechanism and discover new MDR reversal drugs
31849495	Triterpenoid Saponins (TPSs)	Hepatocellular carcinoma (HCC)	Tumor tissues from H22 tumor-bearing mice *in vivo*	GC-TOF-MS	TPSs modulated HCC immune response through regulating the metabolisms of threonine, serine, glycine, beta-alanine, proline, arginine, and histidine	TPSs had the potential in HCC therapy by regulating various signaling cascades related to tumor metabolism and tumorigenesis
29162930	Ku-jin tea (KJT)	Colorectal cancer (CRC)	Urine from AOM-induced CRC rat model *in vivo*	UPLC-QTOF-MS	The purine and amino acid metabolisms were regulated by KJT treatment	The metabolic regulation contributed to the anti-CRC effects of KJT
27443884	American ginseng (AG)	Colon cancer (CC)	Serum and stool from AOM/DSS-induced CC mice model *in vivo*	16S rRNA sequencing and GC-TOF-MS	The metabolisms of amino acids, lipids, and carbohydrates were regulated by AG	AG inhibited CC via reversing the metabolome and microbiome accordingly
30926487	*Z. jujuba* Mill. polysaccharides (ZMPs)	Colorectal cancer (CRC)	Stool from AOM/DSS-induced CRC mice *in vivo*	16S rDNA sequencing and UHPLC-MS	ZMPs intake elevated the levels of total SCFAs and mediated gut microbiota in feces	Close correlations were revealed between wave-shaped metabolites and intestinal flora
30583518	Navy beans	Colorectal cancer (CRC)	Stool from CRC survivors	GC-MS and UPLC-MS/MS	Caprylate, 4-hydroxyphenylacetate, hydantoin-5 propionic acid, and cadaverine were identified in the stool of CRC survivors consuming navy beans, which were related to the microbial metabolism of fatty acids and amino acids.	The acute response of these metabolites and metabolism pathways to long-term consumption of navy bean merited to be further investigation elucidated for ameliorating colonic health
26136108	American ginseng (AG)	Colon cancer (CC)	Serum from a genetically engineered Apc (Min/+) mouse model with high fat diet-enhanced colorectal carcinogenesis *in vivo*	GC-TOFMS and LC-TOFMS	AG treatment greatly altered the levels of linolelaidic acid, arachidonic acid, docosahexaenoate, fructose, glutamate, and tryptophan, all of which were related to inflammation and oxidation	AG showed antineoplastic effects against colon cancer by anti-inflammatory and antioxidant mechanisms
31340453	*D. officinale* Kimura and Migo polysaccharides (DOPs)	Gastric cancer (GAC)	Serum from SD rats of 1-methyl-2-nitro-1-nitrosoguanidine-induced GAC *in vivo*	UPLC/Q-TOF-MS	DOPs significantly altered the level of betaine, which possessed strong antioxidant activity	DOPs could suppress GAC *via* mediating Wnt/β-catenin pathway and altering endogenous metabolites

**TABLE 3:** The summary of recent metabolomics investigations on anti-GIC therapies of formulae from medicinal plants.

References	Formulae from medicinal plants	Cancer types	Samples	Methods	Significantly changed metabolites or pathways	Main findings
31341492	Modified Si Jun Zi Tang (MSJZT)	Gastric cancer (GAC)	Plasma from GAC tumor-bearing nude mice *in vivo*	HILIC UHPLC-Q-TOF/MS	MSJZT treatment could partially reverse the fluctuations in glycolytic, lipid, and amino acid metabolisms	These results provided a basis for further investigation of the precise mechanism of anti-GSC activity of MSJZT
29330507	Kushen injection (KSI)	Hepatocellular carcinoma (HCC)	SMMC-7721 cells *in vitro*	Network analysis and ^1^H-NMR	The regulation of glycometabolism and amino acid metabolism by KSI treatment contributed to its anti-HCC effects	The network pharmacology prediction and metabolomics experimental validation provided a novel insight into the anti-HCC mechanism of KSI
28108381	Shuihonghuazi formula (SHHZF)	Hepatocellular carcinoma (HCC)	Plasma from DEN-induced HCC rat model *in vivo*	HPLC/ESI-TOF-MS	SHHZF treatment increased the shift of PE to PC, linoleic acid metabolism and suppressed the bile acid metabolism	SHHZF may achieved its anti-HCC property *via* reversing the abnormal metabolisms of fatty acids and bile acids
33790982	Jianpi yangzheng xiaozheng (JPYZXZ)	Gastric cancer (GAC)	Serum from GAC patients with chemotherapy	GC-TOF-MS	JPYZXZ reversed the metabolism deficiency of L-valine, L-alloisoleucine, L-leucine, and L-glutamine	JPYZXZ could decrease the adverse drug reactions following chemotherapy and ameliorate the life quality of GAC patients
33129117	*A. mongholicus* Bunge*-C. aromatica* Salisb. (AC)	Colon cancer (CC)	Serum from orthotopic transplantation CC mice model *in vivo*	UPLC-Q-TOF-MS	AC with the ratio of 2:1 exhibited pronounced callback effects on metabolic biomarkers, such as 7-methylxanthine, xanthosine, hypoxanthine, dihomo-γ-linolenic acid, and all-trans-retinoic acid.	AC could partially modulate the metabolic profile to rebalance the metabolic disorder of CC mice, which contributed to the inhibition of proliferation and metastasis of CC, especially at the ratio of 2:1.
29435126	Aidi injection (ADI)	Colorectal cancer (CRC)	Plasma from DMH-induced CRC rat model *in vivo*	UHPLC-MS/MS	The polyamine metabolism, especially putrescine, and agmatine, was significantly reversed after ADI treatment	Plasma polyamine could be a biomarker for both early diagnosis and therapeutics of CRC

## Applications of Metabolomics for Medicinal Plants in Gastrointestinal Cancer

### Carbohydrate Metabolism

Normal cells predominantly acquire energy through mitochondrial oxidative phosphorylation (OXPHOS). However, it is glycolysis rather than OXPHOS by which cancer cells predominantly tend to obtain energy even in the presence of abundant oxygen. This is a famous phenomenon known as “Warburg effect” in cancer (Honigova et al., 2022). Although glycolysis is less efficient than OXPHOS with regard to the production of adenosine triphosphate (ATP), it results in a high abundance of additional metabolites which are beneficial to the highly proliferating cancer cells. Besides, the tricarboxylic acid (TCA) cycle intermediates such as a-ketoglutarate, fumarate, and succinate, are also implicated in cancer initiation and identified as possible biomarkers for cancer drug discovery (Eniafe and Jiang, 2021). In general, carbohydrate metabolism plays a critical role in the tumorigenesis and progression of GIC, which could be a potential therapeutic target for GIC therapy. Various active compounds, extracts, and formulae from medicinal plants targeting carbohydrate metabolism for GIC therapy are obtaining numerous attentions.

As an isoquinoline alkaloid derived from the seeds of *Mucuna pruriens* (L.) DC., 6,7-dimethoxy-1,2,3,4-tetrahydro-isoquinoline-3-carboxylic acid (M1) was reported to inhibit CRC. Based on a ^1^H NMR metabolomics analysis of serum from dimethylhydrazine (DMH)-induced CRC rat model, the disordered metabolic fluxes in CRC condition, including glycolysis, TCA cycle, gluconeogenesis and phosphatidylinositol metabolisms, were reversed after M1 treatment through the oncogenic signalling inhibition of IL-6/JAK2/STAT3 (Mishra et al., 2018). As a bioactive substance isolated from the medicinal plant *Dichroa febrifuga* Lour., halofuginone (HF) was also reported to inhibit CRC. To investigate the underlying mechanism, a combined analysis of GC-MS and UPLC-MS-based metabolomics was performed on HCT116 cells (Chen et al., 2015). In detail, HF treatment decreased both glycolysis and TCA cycle fluxes through Akt/mTORC1 signaling pathways. Koningic acid (KA) is a bioactive compound isolated from *Trichoderma* fungus, which was shown to have anti-CRC potential. Based on an integrated analysis of LC-MS metabolomics and pharmacogenomics on HCT116 cells, KA treatment was found to inhibit CRC *via* suppressing glyceraldehyde 3-phosphate dehydrogenase (GAPDH)-mediated glycolysis, revealing that targeting glucose metabolism may be a potential therapy for CRC (Liberti et al., 2017). As a kind of acyclic diterpenoid from medicinal plants such as *Curcuma longa* L., geranylgeranoic acid (GGA) was reported to trigger cell death of human hepatoma-derived HuH-7 cells. A UPLC-TOF-MS-based metabolomics analysis was conducted to investigate the underlying mechanism how GGA worked against hepatoma (Iwao and Shidoji, 2015). The results showed that GGA may shift HuH-7 cells from glycolysis to mitochondrial OXPHOS by declining fructose 1,6-diphosphate and increasing fructose 6-phosphate. Zerumbone is a sesquiterpene isolated from the medicinal plant *Zingiber zerumbet* (L.) Roscoe ex Sm. with potential anti-HCC property. Wani et al. (2018) conducted a combined analysis of microarray and 2D NMR using Huh7, HepG2 and MHCC-LM3 cells and observed that zerumbone inhibited glycolysis by reducing glucose consumption and lactate production to suppress HCC. As the main active substance also derived from the medicinal plant *C. longa* L., curcumin was shown to be a potential anti-HCC drug. A GC-MS-based metabolomics analysis of the serum from diethylnitrosamine (DEN)-induced HCC mice was performed to study the underlying mechanism (Qiu et al., 2017). In detail, curcumin increased the levels of glucose and fructose as well as decreased the levels of proline and glycine, which protected the mice from the chemical induced liver injury. Li et al. (2016) conducted a UPLC-QTOF-MS-based metabolomics analysis of urine samples of the H22 cell bearing mice to evaluate the anti-HCC effects of hispidulin, which is widely found in various medicinal plants, such as *Artemisia vulgaris* L., *Arnica montana* L., *Eupatorium littorale* Cabrera, and so on. They found that most of the potential biomarkers mediated by hispidulin were associated with TCA cycle and these potential biomarkers underpinned the metabolic pathways, which were perturbed in HCC mice model (Li et al., 2016). In our study, based on a GC-MS metabolomics analysis, we found that berberine, a naturally occurring alkaloid derived from medicinal plant *Coptis chinensis* Franch., exhibited antineoplastic activity against HCC (Guo et al., 2020b). In detail, berberine suppressed the alanine-glucose conversion *via* glutamic-pyruvic transaminase 1 (GPT1) and blocked ATP production and thus inhibited HCC growth. Physapubenolide (PB) derived from medicinal plant *Physalis pubescens* L. is a kind of withanolide. Ma et al. (2016) performed a GC-MS-based metabolomics analysis of HepG2 cells, tumor and plasma from a mouse-xenograft model bearing H22 cells. They found that PB showed anti-HCC activity through suppression of glycolysis *via* the Akt-p53 pathway (Ma et al., 2016). As a quinone-containing component isolated from medicinal plant *Handroanthus impetiginosus* (Mart. ex DC.) Mattos in South America, β-lapachone exhibits anti-pancreatic ductal adenocarcinoma (PDAC) potential. A combined analysis of ^1^H NMR and GC-MS-based metabolomics was conducted on MiaPaCa2 cells to study the underlying mechanism (Silvers et al., 2017). A decrease of nicotinamide adenine dinucleotide-sensitive pathways, such as TCA cycle and glycolysis, was found after β-lapachone treatment. Except from the anti-HCC activity mentioned above, berberine was also observed to inhibit PCC based on an integrated RNA-sequencing and LC-TQ-MS metabolomics analysis of Panc-1 cells and hTERT-HPNE cells (Liu et al., 2020). In detail, berberine strongly dysregulated the energy metabolism of PCC cells *via* targeting citrate metabolism.

Besides the active compounds isolated from medicinal plants targeting carbohydrate metabolism for GIC treatment, there are also various extracts and formulae from medicinal plants targeting carbohydrate metabolism for the therapy of GIC. *Camellia nitidissima* C.W.Chi (CNC) is a kind of rarest plants with anti-CRC property. However, due to its complex components, the anti-CRC efficacy of CNC remains to be explored. Based on a ^1^H NMR metabolomics analysis of the spleen, kidney, and intestine from azoxymethane (AOM)/dextran sodium sulfate (DSS)-induced CRC mice, the treatment of CNC extracts inhibited CRC through restoring the disordered metabolic profiling to the normal status, such as glycolysis, glutamate, and glutamine metabolisms. Moreover, the butanol fraction exhibited a better efficacy against CRC than the water-soluble fraction of CNC (Li et al., 2017). As a characterized extract from the seeds of *Silybum marianum* (L.) Gaertn., silymarin was reported to be a potential anti-hepatoma herb extract. To study the underlying mechanism, Lovelace et al. (2015) performed a combined analysis of GC-MS-based metabolomics and transcriptional profiling on Huh7TLR3 cells. Silymarin suppressed the glycolysis, TCA cycle, and amino acid metabolism, revealing that the attenuated metabolic disorders by silymarin contributed to its antineoplastic activity against hepatoma. The anti-HCC activity was shown in *Rhizoma Paridis* Saponins (RPSs), the active ingredients of the rhizome of *Paris yunnanensis* Franch., called *R. Paridis*. To study the anti-HCC mechanism of RPSs, a ^1^H NMR-based metabolomics analysis of the serum from HCC mice model bearing H22 cells was conducted (Qiu et al., 2016). In detail, RPSs diminished the serum levels of glutamine, N-acetyl amino acid, acetate, and lactate, revealing that RPSs were potential anti-HCC herb extracts *via* suppressing the glutamine metabolism, lipogenesis, and aerobic glycolysis. Si Jun Zi Tang consisting of four medicinal plants, namely *Glycyrrhiza uralensis* Fisch., *Poria cocos* (Schw.) Wolf, *Panax ginseng* C.A.Mey., and *Atractylis macrocephala* Desf., is a famous Chinese herbal formulation. Modified Si Jun Zi Tang (MSJZT) is derived from Si Jun Zi Tang with the addition of two other medicinal plants (*Hedyotis diffusa* Willd. and *C. chinensis* Franch.). Recent a HILIC UHPLC-Q-TOF/MS-based metabolomics of plasma from GAC tumor-bearing nude mice was conducted to investigate the anti-GAC property of MSJZT (Nie et al., 2019). The results showed that MSJZT treatment could partially reverse the fluctuations in glycolytic, lipid, and amino acid metabolisms and thus inhibit GAC. As a famous formulation consisting of two medicinal plants *Sophora flavescens* Aiton and *Heterosmilax japonica* Kunth, Kushen Injection (KSI) is widely utilized to treat various solid tumors. A combined analysis of ^1^H-NMR-based metabolomics and network pharmacology was conducted on SMMC-7721 cells to study the anti-HCC mechanism of KSI (Gao et al., 2018). In detail, the major bioactive ingredients, involved pathways and targets were identified by network pharmacology, which were further confirmed by metabolomics. Remarkably, the regulation of glycometabolism and amino acid metabolism by KSI treatment contributed to its anti-HCC effects.

### Lipid Metabolism

Besides the carbohydrate metabolism playing a vital role in the tumorigenesis and progression of GIC, the lipid metabolism has also been shown to play an essential role *via* regulating the cell membrane synthesis and the essential signalling molecules in fleetly proliferating cancer cells (Yu et al., 2020). For example, choline is a potential biomarker in tumor promotion, which is involved in phospholipid synthesis. Phosphatidylcholine (PC) as the intermediate involved in lipid metabolism, is essential elements for cell membrane. Lysophosphatidylcholine (LysoPC) is the lipid metabolite produced from cytomembrane-derived lipoprotein or PC, which plays a vital role in immunoregulation. The perturbations of these lipid metabolites are associated with GIC oncogenesis. Novel therapeutics for GIC treatment could be provided through targeting the lipid metabolism. In this section, the recent metabolomics investigations of active compounds, extracts, and formulae from medicinal plants targeting lipid metabolism for GIC therapy were reviewed.

As an active component isolated from the soft coral *Sinularia flexibilis*, flexibilide was shown to have potential anti-colon cancer (CC) property. Gao et al. (2016) performed a UPLC-QTOF-MS-based metabolomics analysis of HCT-116 cells to investigate the therapeutic effects of flexibilide on CC. In detail, the significant decrease of phosphocholine and PC and increase of LysoPC were observed after flexibilide treatment, suggesting that the downregulation of PC biosynthesis pathway contributed to the therapeutic effects of flexibilide against CC. As a bioactive compound isolated from the bulbs of medicinal plant *Fritillaria thunbergii* Miq., peiminine exhibits significant anti-CRC activity. Based on a combined analysis of UPLC-MS and GC-MS metabolomics on HCT-116 cells, several metabolites, including oleate (18:1n-9), lignocerate (24:0), glucose, and glutamine were observed to alter after peiminine treatment, revealing that the regulation of lipids, carbohydrates, and amino acids contributed to the therapeutic effects of peiminine on CRC (Zheng et al., 2017). Ilexgenin A (IA) is the primary active compound derived from medicinal plant *Ilex hainanensis* Merr. with pronounced hypolipidemic activity. Recently, its anti-CRC activity has been suggested. Zhang et al. (2019) conducted a UHPLC-Q-TOF-based metabolomics analysis of plasma from AOM/DSS-induced CRC mice model to estimate the effects of IA on CRC and explore its action mechanism. It was found that IA reversed phospholipid metabolism, glycerophospholipid catabolism, and arachidonic acid metabolism through HIF-1α/SREBP-1, revealing that lipid metabolism may be associated with the antineoplastic effects of IA on CRC. Psoralen is a kind of biologically important coumarin derived from the fruits of *Psoralea corylifolia* L., which has been shown to reverse the multidrug-resistance (MDR) of HCC (Thakur et al., 2020). However, the low solubility and poor stability of psoralen limit its wide use. Psoralen packed in polymer lipid nanoparticles (PSO-PLNs) are extensively utilized in drug delivery to increase the solubility and stability of psoralen. To evaluate the beneficial effects of PSO-PLNs against HCC, a LC-MS-based metabolomics analysis was conducted on serum and urine from mice engrafted with HepG2 cells resistant against doxorubicin (Li et al., 2021). In detail, PSO-PLNs had stronger degree of regression of metabolites including α-linolenic acid, retinoic acid, pantothenic acid, hyaluronic acid, and hippuric acid than psoralen. Psoralen reversed drug resistance of HCC cells, which could be enhanced by encapsulation in polymer lipid nanoparticles.

Besides the active compounds isolated from medicinal plants mentioned above, some extracts and formulae from medicinal plants targeting lipid metabolism for GIC therapy have also been reported. As a seed of the fruit of medicinal plant *Myristica fragrans* Houtt., nutmeg has various pharmacological activities, including anti-CC. Based on a UPLC-ESI-QTOF-MS-based metabolomics analysis of serum from CC mice model, the lipid metabolism was regulated by the treatment of nutmeg extract *via* diminishing four uremic toxins formed from the gut microbiota, suggesting a close relationship between lipid metabolism and gut microbiota in the effective anti-CC effects of nutmeg (Li et al., 2015). Foxtail millet is one of the important cereals in China and peroxidase from foxtail millet bran (FMBP) exhibits beneficial effects against CRC. Shan et al. (2020) conducted a UPLC-Triple/TOF-MS-based metabolomics analysis of serum from AOM/DSS-induced colitis-associated carcinogenesis mice model to investigate the underlying mechanism. In detail, FMBP predominantly decreased the levels of PC and phosphatidylethanolamine (PE) related to glycerophospholipid (GPL) metabolism, which was a potential preventive and therapeutic extract to blockade the GPL metabolism for CRC. *Bornean Sinularia* sp. (BS) has been shown to inhibit HCC. A MS-based metabolomics analysis of Hep 3B cells was performed to study the inhibitory mechanisms of BS extract to HCC (Ling et al., 2020). The levels of ceramide and sphingolipids were regulated by BS extract, indicating that BS showed its anti-HCC property via the regulation of disordered lipids. As a traditional formula made from four herbal medicines, including *Polygonum orientale* L., *Ophicalcitum*, *Imperata cylindrica* (L.) Beauv., and *Coix lacryma-jobi* var. *ma-yuen* (Rom.Caill.) Stapf, Shuihonghuazi Formula (SHHZF) has been clinically used for HCC treatment. A HPLC/ESI-TOF-MS-based metabolomics analysis was conducted on the plasma from DEN-induced HCC rat model to study the underlying mechanism (Bao et al., 2017). It was found that SHHZF treatment increased the shift of PE to PC, linoleic acid metabolism and suppressed the bile acid metabolism, revealing that SHHZF may achieved its anti-HCC property *via* reversing the abnormal metabolisms of fatty acids and bile acids.

### Amino Acid Metabolism

Cancer cells need a large amount of amino acids from the amino acid pool to generate protein and nucleic acid in order to meet their rapid proliferation (Yu et al., 2020). Besides the consumption of glucose as energy source, glutamine is also favoured as a preferential fuel for cancer cells *via* glutaminolysis. In detail, glutamine is converted to glutamate and then converted to α-ketoglutarate, which enters TCA cycle to produce energy. In addition to energy production, glutamine also plays a vital role in devoting the synthesis of lipids and nucleic acids, and mediating redox homeostasis. Glutamine metabolism plays a vital role in the tumorigenesis and progression of GIC and various other amino acid metabolisms have also been shown to be of primary importance in GIC, which could be potential targets for GIC therapy. The recent investigations of active compounds, extracts, and formulae from medicinal plants targeting amino acid metabolism for GIC therapy were reviewed.

As an active component isolated from *Tripterygium wilfordii* Hook.f., celastrol has the potential antineoplastic property against CC. A UPLC-MS-based metabolomics analysis was performed on HCT116 cells to study the underlying mechanism how celastrol worked against CC (Qi et al., 2018). In detail, the levels of lipid markers, amino acids, and carnitine were significantly altered after celastrol treatment and further targeted metabolite analysis characterized tryptophan as the key biomarker. Oridonin is a tetracyclic diterpenoid derived from medicinal plant *Isodon rubescens* (Hemsl.) H.Hara with potential anti-EC activity. To investigate the underlying mechanism, a UPLC-MS/MS-based metabolomics analysis of TE1 cells was conducted. It was found that oridonin administration altered the levels of glutamate, 5-oxoproline, and gamma-glutamyl amino acids (gamma-glutamyl valine and gamma-glutamyl leucine), which could suppress the gamma-glutamyl cycle to induce ferroptosis to exert its anti-EC activity (Zhang et al., 2021).

In addition to active compounds from medicinal plants, the extracts and formulae from medicinal plants have also been reported to show potential inhibitory effects against GIC *via* targeting amino acid metabolism. For example, polyphenols are characterized as hydroalcoholic extracts of chestnut shell with anti-HCC activity. To investigate the anti-HCC activity of polyphenols extracted from chestnut shell (PECS), a ^1^H-NMR-based metabolomics analysis was conducted on HepG2 cells (Sorice et al., 2016). The results revealed that PECS could modulate the levels of some amino acids to inhibit HCC. As a series of polyketides widely isolated from medicinal plant *Annona squamosa* L., annonaceous acetogenins (ACGs) show a wide range of biological properties, including antineoplastic property against HCC. Based on a UFLC-ESI-Q-TOF-MS metabolomics analysis of SMMC 7721 cells, it was found that ACGs treatment could regulate the metabolisms of proline, arginine, glutathione, and sphingolipid, which further reversed the resistance of SMMC 7721 cells to adriamycin, suggesting that metabolic pathway analysis combined with stoichiometry analysis could be a potential tool to understand MDR mechanism and discover new MDR reversal drugs (Ma et al., 2019). Triterpenoid Saponins (TPSs) are a class of active compounds derived from medicinal plant *Anemone flaccida* Fr. Schmidt with multiple bioactive properties. Based on a GC-TOF-MS-based metabolomics analysis of tumor tissues from H22 tumor-bearing mice, Han et al. (2019) found that TPSs modulated HCC immune response through regulating the metabolisms of threonine, serine, glycine, beta-alanine, proline, arginine, and histidine, which had the potential in HCC therapy by regulating various signaling cascades related to tumor metabolism and tumorigenesis. As a famous Chinese herbal formulation, Jianpi Yangzheng Xiaozheng (JPYZXZ) has been widely used in clinic for decades. Previous clinical studies have indicated that JPYZXZ could prolong the survival time and relieve the pain of Chinese GAC patients in advanced stage. To investigate the underlying mechanism, a GC-TOF-MS-based metabolomics analysis of serum from GAC patients with chemotherapy was performed. It was found that JPYZXZ reversed the metabolism deficiency of L-valine, L-alloisoleucine, L-leucine, and L-glutamine, which could decrease the adverse drug reactions following chemotherapy and ameliorate the life quality of GAC patients (Hou et al., 2021).

### Nucleotide Metabolism

It is known that the *de novo* synthesis of purine and pyrimidine is fundamental for cancer cell growth. Cancer cells exhibit altered nucleotide metabolisms, such as nucleic acid synthesis, in order to favour their fast proliferation (Yu et al., 2020). Consequently, novel therapeutics for GIC treatment *via* targeting nucleotide metabolism has gained great interest. In this section, the recent metabolomics investigations of active compounds, extracts, and formulae from medicinal plants targeting nucleotide metabolism in GIC were reviewed.

Several active compounds from medicinal plants have been reported to have potential inhibitory effects on GIC *via* targeting nucleotide metabolism. For instance, shikonin is a naphthoquinone compound derived from the root of *Lithospermum erythrorhizon* Siebold & Zucc. with potential anti-CC potency. To investigate the underlying mechanism how shikonin worked against CC, an integrated analysis of transcriptomics and UHPLC-MS-based metabolomics was conducted on SW480 cells. Shikonin intervention caused significant fluctuations of a total of 1642 genes and 40 metabolites. The integrated analysis revealed that the regulation of purine and pyrimidine metabolisms, urea cycle, and arginine biosynthesis contributed to the anti-CC activity of shikonin (Chen et al., 2021). As an ent-kaurene diterpenoid isolated from *Rabdosia japonica* (Burm.f.) H.Hara, glaucocalyxin A (GLA) was reported to be a potential anti-HCC agent. A combined LC-MS and GC-MS-based metabolomics analysis of SMMC7721 cells was conducted to study the underlying mechanism (Liu et al., 2018b). In detail, GLA treatment suppressed amino acid metabolism and increased the metabolisms of sphingolipid, pyrimidine, and purine, which contributed to the anti-HCC activity of GLA.

In addition to active compounds from medicinal plants, several extracts and formulae from medicinal plants were also reported to exhibit potential inhibitory effects on GIC *via* targeting nucleotide metabolism. For example, as a classic beverage isolated from the leaves of the medicinal plant *Acer tataricum subsp. ginnala* (Maxim.) Wesm., Ku-jin tea (KJT) shows potential anti-CRC activity. To study the therapeutic role of KJT in CRC, a UPLC-QTOF-MS-based metabolomics analysis was performed on the urine from AOM-induced CRC rats (Bi et al., 2017). In detail, the purine and amino acid metabolisms were regulated by KJT treatment, which contributed to the anti-CRC activity of KJT. *Astragalus mongholicus* Bunge-*Curcuma aromatica* Salisb. (AC) is a drug pair with potential antineoplastic activity against CC. Sun et al. (2021) conducted a UPLC-Q-TOF-MS-based metabolomics analysis of serum from orthotopic transplantation CC mice model to explore the best ratio of AC and investigate how AC worked against CC. In detail, AC with the ratio of 2:1 exhibited pronounced callback effects on metabolic biomarkers, such as 7-methylxanthine, xanthosine, hypoxanthine, dihomo-γ-linolenic acid, and all-trans-retinoic acid. AC could partially modulate the metabolic profile to rebalance the metabolic disorder of CC mice, which contributed to the inhibition of proliferation and metastasis of CC, especially at the ratio of 2:1.

### Other Related Metabolisms

In addition to the anti-GIC therapies of medicinal plants *via* targeting the metabolisms mentioned above, several other related metabolisms such as microbial metabolism, inflammation and oxidation have also been reported to be associated with the therapeutic role of medicinal plants in GIC.

Multiple lines of evidences have suggested that gut microbiota, as a new functional organ, could mediate microbial metabolism to generate a body of compounds, including vitamin K, indole, and fatty acids, which contribute to the tumorigenesis of GIC, especially for CRC (Nannini et al., 2020). The stool metabolome contains various metabolites associated with nutrient ingestion, digestion and absorption by gastrointestinal tract and gut microbiota, such as undigested food residues (mucopolysaccharides, fiber, etc.) and small molecules (amino acids, organic acids, and sugars, etc.) (Baxter et al., 2018). Medicinal plants targeting microbial metabolism for GIC therapy are gaining momentum. For instance, based on a combined analysis of 16S rRNA sequencing and ^1^H NMR metabolomics using stool from AOM/DSS-induced CRC mice model, Chen et al. (2020) found that berberine induced changes in metabolome and microbiota in CRC, which could provide a novel insight into the anti-CRC effects of berberine. In detail, berberine increased some short-chain fatty acids (SCFA)-producing bacteria, including *Oscillibacter*, *Flavonifractor*, and *Alloprevotella*, and inhibited the pathogenic species, such as *Alistipes* and *Erysipelotrichaceae*. Metabolic data further revealed that berberine mediated metabolic fluctuations in feces *via* regulating the amino acid metabolism, SCFA metabolism, and glycometabolism, which were the products of interactions between the host and gut microbiota (Chen et al., 2020). Caffeic acid (CAA) and chlorogenic acid (CHA) are both in the kind of polyphenol derived from *Lonicera japonica* Thunb. with anti-HCC properties. A combined analysis of 16S rRNA sequencing, LC-MS and GC-MS-based metabolomics was performed on the serum from DEN-induced HCC rat model to study the molecular mechanism (Zhang et al., 2017). Both CAA and CHA treatments reversed 28 metabolites. In detail, the levels of bilirubin, ethanolamine, L-tyrosine, and L-methionine were linked to the increased level of *Rumincoccaceae UCG-004* and decreased levels of *Lachnospiraceae incertae sedis* and *Prevotella 9*. Ren et al. (2020) developed a novel nanoparticle with ginsenoside Rg3 (NpRg3), which is a kind of steroidal saponin derived from *P. ginseng* C.A.Mey. Based on an integrated analysis of 16S rRNA sequencing and HPLC-MS metabolomics, they found that NpRg3 administration increased the richness of *Verrucomicrobia* and *Bacteroidetes*, but decreased *Firmicutes*. Moreover, NpRg3 treatment increased free fatty acids, but diminished urea and 3-indolepropionic acid to remodel the network imbalance between metabolism and gut microbiota, which contributed to inhibit HCC development and metastasis (Ren et al., 2020). American ginseng (*Panax quinquefolius* L., AG) is a famous medicinal plant, which is widely used for the treatment of CC. To explore the detailed response of CC to AG, a combined analysis of 16S rRNA sequencing and GC-TOF-MS-based metabolomics was conducted on the serum and stool from AOM/DSS-induced CC mice (Wang et al., 2016). The 16S rRNA data showed that AG inhibited AOM/DSS-induced changes in microbiome community. Besides, the metabolisms of amino acids, lipids, and carbohydrates were regulated by AG and AG inhibited CC *via* reversing the metabolome and microbiome accordingly. An integrated analysis of 16S rDNA sequencing and UHPLC-MS-based metabolomics on stool from AOM/DSS-induced CRC mice was performed to investigate the anti-CRC property of *Ziziphus jujuba* Mill. polysaccharides (ZMPs) (Ji et al., 2019). It was found that ZMPs intake mediated gut microbiota via increasing the abundance of *Lactobacillus*, *Bacteroides*, and *Bifidobacterium* and elevated the levels of total SCFAs in feces. Based on a combined analysis of GC-MS and UPLC-MS/MS metabolomics on stool from CRC survivors, Baxter et al. (2018) found that caprylate, 4-hydroxyphenylacetate, hydantoin-5 propionic acid, and cadaverine were identified in the stool of CRC survivors consuming navy beans, which were related to the microbial metabolism of fatty acids and amino acids and contributed to ameliorate colonic health.

Chronic inflammation and oxidation have been shown to play an essential role in tumorigenesis and progression of GIC (Dai et al., 2022), which could be the potential targets of medicinal plants for GIC therapy. For instance, solasonine is an active compound derived from *Solanum melongena* L. with potential antineoplastic activity against HCC. Jin et al. (2020) conducted a LC-MS-based metabolomics analysis of HepG2 cells and found that solasonine induced HCC cell ferroptosis by the destruction of the glutathione redox system. A combined analysis of GC-TOFMS and LC-TOFMS-based metabolomics on serum from CC mice model revealed that AG treatment greatly altered the levels of linolelaidic acid, arachidonic acid, docosahexaenoate, fructose, glutamate, and tryptophan, all of which were related to inflammation and oxidation (Xie et al., 2015). *Dendrobium officinale* Kimura & Migo polysaccharides (DOPs) are the primary bioactive ingredients derived from medicinal plant *D. officinale* Kimura & Migo. Zhao et al. (2019) conducted a UPLC/Q-TOF-MS-based metabolomics analysis on serum from GAC rat model and found that DOPs significantly altered the level of betaine, which possessed strong antioxidant activity and contributed to the anti-GAC effects of DOPs.

Besides microbial metabolism, inflammation and oxidation mentioned above, there are also some other metabolisms which are targeted by medicinal plants for GIC therapy. For example, Aidi Injection (ADI) is a famous Chinese herb injection with potential antineoplastic property against CRC. Based on a UPLC-MS/MS metabolomics analysis of plasma from DMH-induced CRC rat model, it was observed that the polyamine metabolism, especially putrescine and agmatine, was significantly reversed after ADI treatment, identifying plasma polyamine as the potential biomarker for both early diagnosis and therapeutics of CRC (Liu et al., 2018a).

## Current Perspectives and Future Challenges

Medicinal plants are a treasure trove for new drug discovery and development in GIC. However, due to their own distinct theoretical system with multi-component and multi-target manifestation, the underlying mechanisms how medicinal plants work against GIC remain to be fully deciphered. In good agreement with the holistic perspective and overall concept of medicinal plants, metabolomics could provide a novel insight into the exploration of the underlying mechanisms of medicinal plants for GIC therapy. Based on our systematic review, different compounds, extracts and formulae from medicinal plants exhibited specific effects on GIC *via* different metabolism pathways presumably due to diverse chemical structures and multi-component interactions. The potential therapies for GIC with active compounds, extracts, and formulae from medicinal plants targeting cancer metabolism have attracted considerable attentions from the metabolomics perspective. Crucially, the systemic biofluids used for metabolomics, such as blood (serum and plasma), urine, and fecal water are collected in a simple, non-invasive or minimally invasive way and analyzed using a high throughput, robust, quantitative, and reproducible technique, which makes it possible to move the massive basic metabolomics research to translational or even clinical application for GIC therapy.

Although a huge advance has been achieved, great challenges remain to be settled in the metabolomics study of medicinal plants for GIC therapy. One of the biggest challenges occurs in the inconsistency of the relative abundance of same metabolites among different researches. There is still a long way for the widespread use of metabolomics into the mechanism investigation of medicinal plants for GIC therapy. Firstly, before starting the experiments of metabolomics, several steps are critical to be considered, such as the choices of experimental sample species and amounts, analytical chemistry platforms, sample processing procedures, data methodological means and so on, in order to make good experimental design. Secondly, there are only small portions of metabolites which have been exactly identified in current metabolomics. Metabolomics should be largely developed to identify and quantify more metabolites in various aspects, such as analytical chemistry platform development and big database exploitation for deep data excavation. Thirdly, unlike the biomarkers of gene and protein, metabolite biomarker is susceptible to environment such as diet styles. Backgrounds should be considered in the results of metabolomics and the results need further intensive verification procession. Fourthly, no single technology could offer an entire spectrum of cancer. In order to better explore the underlying mechanisms of antineoplastic medicinal plants for GIC therapy, it is remarkably recommended to combine different analytical chemistry platforms and integrate metabolomics with some other advanced technologies, such as the upstream “Omics,” gut microbiome and network pharmacology analyses. Last but not least, due to the complicated interactions between genes and environments (xenobiotics, gut microbiota, and polypharmacy), different responses even to the same drug treatment are observed among cancer patients. Precision medicine, also known as personalized medicine to address cancer is recognized as “precision oncology,” which is put forward and gradually attracting considerable attentions nowadays (Di Nicolantonio et al., 2021). The goal of precision oncology is to use advanced omics testing to customize an individual’s medical treatment to improve the curative effects, avoid side effects and medical resource waste for cancer according to their specific biomarker profiles. Although pharmacogenomics is still the only approach for precision medicine, the environmental influences are usually ignored in pharmacogenomics study. Pharmacometabolomics is a new emerging “omics” science as an alternative and complementary approach, which has been proposed for precision medicine (Mussap et al., 2020). By considering the influences of both genes and environments, pharmacometabolomics could offer individual metabolic profiles of both environment-derived exogenous and gene-derived endogenous metabolites and contribute to the better understanding of individual phenotypic variations in response to drug, which would provide an intriguingly avenue for precision oncology in the future. Despite challenges for the development of metabolomics exist and it is a long way to move the massive basic metabolomics research to translational or even clinical application, we still expect that metabolomics will gain full acceptance in the clinical setting for GIC diagnosis and anti-GIC medicinal plant development.

## Conclusion

In this review, the metabolomics investigations on metabolism-targeting therapies for GIC in the recent 10 years were systematically reviewed at five aspects of carbohydrate, lipid, amino acid, and nucleotide metabolisms, as well as other altered metabolisms (microbial metabolism, inflammation, and oxidation), with particular attention to the potential of active compounds, extracts, and formulae from medicinal plants. Meanwhile, the current perspectives and future challenges of metabolism-targeting therapies of medicinal plants for GIC were also discussed. Hopefully, based on our systematic review on the recent mechanism investigation of medicinal plants for GIC therapy from the metabolomics perspective, more attention would be attracted to the clinical application of potential candidates from the resourceful medicinal plants as novel and efficient adjuvant therapeutics for GIC therapy.
